# Experimental infection of hamsters with avian paramyxovirus serotypes 1 to 9

**DOI:** 10.1186/1297-9716-42-38

**Published:** 2011-02-23

**Authors:** Arthur S Samuel, Madhuri Subbiah, Heather Shive, Peter L Collins, Siba K Samal

**Affiliations:** 1 Virginia-Maryland Regional College of Veterinary Medicine, University of Maryland, College Park, Maryland, USA; 2 Experimental Transplantation and Immunology Branch, National Cancer Institute/National Institute of Health, Bethesda, USA; 3 Laboratory of Infectious Diseases, National Institute of Allergy and Infectious Diseases, Bethesda, USA

## Abstract

Avian paramyxoviruses (APMVs) are frequently isolated from domestic and wild birds throughout the world and are separated into nine serotypes (APMV-1 to -9). Only in the case of APMV-1, the infection of non-avian species has been investigated. The APMVs presently are being considered as human vaccine vectors. In this study, we evaluated the replication and pathogenicity of all nine APMV serotypes in hamsters. The hamsters were inoculated intranasally with each virus and monitored for clinical disease, pathology, histopathology, virus replication, and seroconversion. On the basis of one or more of these criteria, each of the APMV serotypes was found to replicate in hamsters. The APMVs produced mild or inapparent clinical signs in hamsters except for APMV-9, which produced moderate disease. Gross lesions were observed over the pulmonary surface of hamsters infected with APMV-2 & -3, which showed petechial and ecchymotic hemorrhages, respectively. Replication of all of the APMVs except APMV-5 was confirmed in the nasal turbinates and lungs, indicating a tropism for the respiratory tract. Histologically, the infection resulted in lung lesions consistent with bronchointerstitial pneumonia of varying severity and nasal turbinates with blunting or loss of cilia of the epithelium lining the nasal septa. The majority of APMV-infected hamsters exhibited transient histological lesions that self resolved by 14 days post infection (dpi). All of the hamsters infected with the APMVs produced serotype-specific HI or neutralizing antibodies, confirming virus replication. Taken together, these results demonstrate that all nine known APMV serotypes are capable of replicating in hamsters with minimal disease and pathology.

## Introduction

*Paramyxoviridae *is a large and diverse family whose members have been isolated from many species of avian, terrestrial, and aquatic animal species around the world [1]. Paramyxoviruses are pleomorphic, enveloped, cytoplasmic viruses that have a non-segmented, negative-sense RNA genome. The family is divided into two subfamilies, *Paramyxovirinae *and *Pneumovirinae*, based on their structure, genome organization, and sequence relatedness [2]. The subfamily *Paramyxovirinae *contains five genera: *Respirovirus, Rubulavirus, Morbillivirus, Henipavirus*, and *Avulavirus*, while the subfamily *Pneumovirinae *contains two genera, *Pneumovirus *and *Metapneumovirus *[3]. All paramyxoviruses that have been isolated to date from avian species can be segregated into two genera based on the taxonomic criteria mentioned above: genus *Avulavirus*, whose members are called the avian paramyxoviruses (APMV), and genus *Metapneumovirus*, whose members are called avian metapneumoviruses. The APMV of genus *Avulavirus *are separated into nine serotypes (APMV-1 through -9) based on Hemagglutination Inhibition (HI) and Neuraminidase Inhibition (NI) assays [4]. Various strains of APMV-1, which is also called Newcastle disease virus (NDV), have been analyzed in detail by biochemical analysis, genome sequencing, and pathogenesis studies, and important molecular determinants of virulence have been identified [5-9]. As a first step in characterizing the other APMV serotypes, complete genome sequences of one or more representative strains of APMV serotypes 2 to 9 were recently determined, expanding our knowledge about these viruses [10-18].

APMV-1 comprises all strains of NDV and is the best characterized serotype because of the severity of disease caused by virulent NDV strains in chickens. NDV strains vary greatly in their pathogenicity to chickens and are grouped into three pathotypes: highly virulent (velogenic) strains, which cause severe respiratory and neurological disease in chickens; moderately virulent (mesogenic) strains, which cause mild disease; and non-pathogenic (lentogenic) strains, which cause inapparent infections. In contrast, very little is known about the comparative disease potential of APMV-2 to APMV-9 in domestic and wild birds. APMV-2 strains have been isolated from chickens, turkeys and wild birds across the globe [4,19-22]. APMV-2 infections in turkeys have been found to cause mild respiratory disease, decreases in egg production, and infertility [23,24]. APMV-3 strains have been isolated from wild and domestic birds [25]. APMV-3 infections have been associated with encephalitis and high mortality in caged birds [26-28]. APMV-4 strains have been isolated from chickens, ducks and geese [29]. Experimental infection of chickens with APMV-4 resulted in mild interstitial pneumonia and catarrhal tracheitis [30]. APMV-5 strains have only been isolated from budgerigars (*Melopsittacus undulatus*) and cause depression, dyspnoea, diarrhea, torticollis, and acute fatal enteritis in immature budgerigars, leading to very high mortality [31]. APMV-6 was first isolated from a domestic duck and was found to cause mild respiratory disease and drop in egg production in turkeys, but was avirulent in chickens [10,30,32]. APMV-7 was first isolated from a hunter-killed dove and has also been isolated from a natural outbreak of respiratory disease in turkeys. APMV-7 infection in turkeys caused respiratory disease, mild multifocal nodular lymphocytic airsacculitis, and decreased egg production [33]. APMV-8 was isolated from a goose and a feral pintail duck [34,35]. APMV-9 strains have been isolated from ducks around the world [36,37]. APMV types -2, -3, and -7 have been associated with mild respiratory disease and egg production problems in domestic chickens [33]. There are no reports of isolation of APMV-5, -8 and -9 from poultry [32]. But recent serosurveillance of commercial poultry farms in USA indicated the possible prevalence of all APMV serotypes excluding APMV-5 in chickens [38].

APMV-1 (NDV) is known to replicate in non-avian species including humans [39-44], although its only natural hosts are birds. APMV-1 infections in non-avian species are usually asymptomatic or mild. Clinical signs in human infections commonly involve conjunctivitis, which usually is transient and self-limiting. Presently, APMV-1 is being evaluated as a vaccine vector against human pathogens [45]. When administered to the respiratory tract of non-human primates, NDV is highly restricted in replication, but foreign antigens expressed by recombinant NDV vectors are moderately to highly immunogenic. One of the major advantages of this approach is that most humans do not have pre-existing immunity to APMV-1. Pre-existing immunity is a potential drawback to using vectors derived from common human pathogens, and also can be a concern for any vector if two or more doses are necessary to elicit protective immunity. Therefore, we are investigating APMV types 2 to 9, which are antigenically distinct from APMV-1, as alternative human vaccine vectors. Also, some of these additional APMV types likely will have differences in replication, attenuation, and immunogenicity compared to APMV-1 that may be advantageous. However, the replication and pathogenicity of APMV-2 to -9 in non-avian species has not been studied. As a first step, we have evaluated the replication and pathogenicity of APMV-2 to -9 in hamsters. In this study, groups of hamsters were infected with a prototype strain of each APMV serotype by the intranasal route and monitored for virus replication, clinical symptoms, histopathology, and seroconversion. Our results showed that each of the APMV serotypes replicated in hamsters without causing adverse clinical signs of illness, although histopathologic evidence of disease was observed in some cases, and also induced high neutralizing antibody titers.

## Materials and methods

### Viruses and cells

The following nine prototype strains of APMV serotypes 1 to 9 were used, APMV-1, NDV lentogenic strain LaSota/46; APMV-2, APMV-2/Chicken/California/Yucaipa/56; APMV-3, APMV-3/PKT/Netherland/449/75; APMV-4, APMV-4/duck/HongKong/D3/75; APMV-5, APMV-5/budgerigar/Kunitachi/74; APMV-6, APMV-6/duck/HongKong/18/199/77; APMV-7, APMV-7/dove/Tennessee/4/75; APMV-8, APMV-8/goose/Delaware/1053/76; APMV-9, APMV-9/duck/New York/22/1978. All of the viruses were grown in 9-day-old embryonated specific-pathogen-free (SPF) chicken eggs inoculated by the allantoic route, except for APMV-5, which was grown in African green monkey kidney (Vero) cells (ATCC, Manassas, VA, USA) [17]. The allantoic fluids from infected eggs were collected 96 h post-inoculation and virus titers were determined by hemagglutination (HA) assay with 0.5% chicken RBC except in the case of APMV-5, which was titrated by plaque assay on Vero cells. The APMV-5 samples were inoculated in triplicate onto 24-well plates of Vero cells at 80% confluency, incubated for 1 h, washed with PBS, overlaid with 0.8% methylcellulose, and observed for plaque production till 7 days post infection (dpi). The cells were fixed with methanol and stained with 1% crystal violet. Values for each tissue sample were based on average plaque count from three wells. Vero cells were grown in Earle's minimum essential medium (EMEM) with 10% fetal bovine serum (FBS). Chicken embryo fibroblast (DF-1) cells were grown in Dulbecco's minimal essential medium (DMEM) containing 10% FBS at 37°C with 5% CO_2_.

### Preparation of hyperimmune antisera against viral nucleocapsid (N) proteins in rabbits

For each of the prototype strains, virions were purified on discontinuous sucrose gradients [12]. The viral proteins were denatured and reduced and were separated on 10% SDS-Polyacrylamide gels and negatively stained using E-Zinc ™reversible stain kit (Pierce, Rockford, IL, USA). Each serotype was analyzed on a separate gel to avoid cross contamination. The N protein bands were excised from the gels and destained with Tris-glycine buffer, pH 8. The excised gel bands were minced in a clean pestle and mixed with elution buffer (50 mM Tris-HCl buffer pH 8, 150 mM NaCl, 0.5 mM EDTA, 5 mM DTT and 0.1% SDS) and transferred to the upper chambers of Nanosep centrifugal device (Pall Life Sciences, Ann Arbor, MI, USA). The proteins were eluted by centrifugation at 13000 × *g *for 5 minutes, and the eluted proteins were quantified and 200 μg of each protein was mixed in complete Freund's adjuvant and injected subcutaneously into a rabbit. After two weeks, a booster immunization was given with 200 μg of protein in incomplete Freund's adjuvant and two weeks later the hyperimmune sera were collected. The sera were tested by western blot and were found to recognize specifically the homologous N protein (data not shown). The sera were stored at -80°C until further use.

### Experimental infection of hamsters

To study viral replication and pathogenicity, fifty seven 4-week-old Syrian golden hamsters (Charles River Laboratories Inc, Wilmington, MA, USA) were housed in negative-pressure isolators under Bio Safety Level (BSL)-2 conditions and provided feed and water ad libitum. The animals were cared for in accordance with the Animal Welfare Act and the Guide for Care and Use of Laboratory Animals and the protocols were approved by the institution's IACUC. Hamsters in groups of six were inoculated intranasally with 100 μL of infectious allantoic fluid containing 2^8 ^HAU of each individual APMV, except for APMV-5, which contained 3 × 10^3 ^PFU/mL, under isoflurane anesthesia. A group of three hamsters served as uninfected controls and were mock infected with normal allantoic fluid. The hamsters were observed three times daily for physical activity and for any clinical signs of illness, and were weighed on day 0, 5 and 14 dpi. Three hamsters from each group were euthanized at 3 dpi and the other three (as well as the three animals in the control group) at 14 dpi by rapid asphyxiation in a CO_2 _chamber. Necropsies were performed immediately postmortem and the following tissue samples were collected for immunohistochemistry (IHC), histopathology, and virus isolation: brain, nasal turbinates, lung, spleen, kidney and small intestine. In addition serum samples were collected on 14 dpi immediately prior to euthanasia, and seroconversion was evaluated by HI assay [46].

### Virus detection and quantification from tissue samples

Half of each tissue sample was used for virus titration. These samples were collected aseptically in 1 mL of DMEM in 10X antibiotic solution containing 2000 units/mL penicillin, 200 ug/mL gentamicin sulfate, and 4 ug/mL amphotericin B (Sigma chemical co., St. Louis, MO, USA). They were processed immediately to avoid any reduction in virus titers. Briefly, a 10% homogenate of the tissue samples were prepared by using a homogenizer and clarified by centrifugation at 420 × *g *for 10 min. For all of the serotypes except APMV-5, the virus titers in the clarified supernatants were determined by end-point dilution on DF-1 cells. Tenfold dilutions of tissue supernatant were inoculated onto DF-1 cells in 96-well plates and incubated for three days. The plates were fixed in 10% formalin for 30 min and the cells were permeabilized using 2% Triton X-100 for 2 min. The plates were washed five times with PBS to remove any residual formalin in the wells. The cells were blocked using 2% normal goat serum for 60 min and the plates were washed twice with PBS- Tween-20 (PBS-T). The cells were incubated with primary rabbit antiserum raised against the N protein of the homologous APMV serotype at room temperature for 1 h. The plates were washed with PBS twice, 5 min each. The cells were incubated with anti rabbit FITC antibody as secondary antibody for 45 min and washed finally with PBS twice, 5 min each. The slides were visualized and the virus titers were determined by end point titration method using the Reed and Muench formula, and were photomicrographed using a fluorescent microscope (Zeiss Axioshop 2000, Zeiss, Göttingen, Germany) [47]. The virus titers in the supernatants of tissue homogenates from APMV-5 infected hamsters were determined by plaque assay in Vero cells as described above using 10-fold dilution series. Values for each tissue sample were based on average plaque count from three wells.

### Immunohistochemistry (IHC) and histopathology

The other half of each tissue sample was used for IHC and histopathology. The tissues were fixed in 10% neutral buffered formalin, held for approximately seven days, and processed for IHC and histopathology. Paraffin embedded 5-micron sections of all the tissue samples were prepared at Histoserve, Inc. (Maryland, MD, USA). The sections were stained with hematoxylin and eosin for histopathology. Sections were also immunostained to detect viral N protein using the following protocol. Briefly, the tissue sections were deparaffinized in two changes of xylene for 5 min each, hydrated in two changes of 100% ethanol for 3 min each, changes of 95% and 80% ethanol for 1 min each, and finally washed in distilled water. The sections were processed for antigen retrieval in a water bath containing sodium citrate buffer (10 mM citric acid, 0.05% Tween 20, pH 6.0), at 95-100°C for 40 min and then allowed to cool to room temperature for another 60 min. The sections were rinsed in PBS-Tween 20, twice for 2 min each. The sections were blocked with 2% BSA in PBS for 1 h at room temperature. The sections were then incubated with a 1:500 dilution of the homologous primary N-specific rabbit antiserum in PBS for 1 h in a humidified chamber. After three washes in PBS, the sections were incubated with the secondary antibody (FITC conjugated goat anti-rabbit antibody) for 30 min. After a further wash cycle, the sections were mounted with glycerol and viewed under an immunofluorescence microscope.

### Serological analysis

Sera were collected from all the hamsters on 14 dpi and evaluated for seroconversion by HI assay [46], except in the case of APMV-5. In addition, cross-HI tests were performed to investigate cross reactivity with other APMV serotypes. Since APMV-5 does not cause hemagglutination of chicken RBC, the antibody titer was determined by plaque reduction neutralization assay. Briefly, the sera were heat inactivated at 56°C for 30 min. Ten-fold dilutions of sera were made and mixed with a constant amount of APMV-5 (3 × 10^3 ^PFU), and incubated at room temperature for 1 h in a shaker. The antigen antibody mixtures were analyzed by plaque assay as described above. The serum titer that reduced plaque numbers by 70% was the end point titer.

## Results

### Clinical disease and gross pathology

Four-week-old hamsters in groups of six were inoculated by the intranasal route with 2^8 ^HA units of each of the APMV serotypes except APMV-5, which was administered at a dose of 3 × 10^3 ^PFU/mL. Three animals were sacrificed at day 3 and the remaining three animals were sacrificed on day 14; following sacrifice, the animals were processed for gross pathology, histopathology, IHC, quantitative virology, and seroconversion. Three uninfected animals served as controls and were sacrificed and processed on day 14. All of the animals were observed three times daily and weighed daily. None of the hamsters infected with any of the APMV serotypes showed any visible clinical signs of disease except those infected with APMV-9. All the six hamsters infected with APMV-9 serotype were dull and had rough skin coat at 3 dpi. They appeared weak and lost weight (Table 1) till 5 dpi, but later gained body weight and appeared normal by 10 dpi. None of the hamsters died of disease. The uninfected control hamsters appeared healthy and normal.

**Table 1:** Body weights (in grams) of hamsters infected with APMV serotypes 1 to 9, measured on 0, 5 and 14 days post inoculation (dpi).

APMV	0 dpi	5 dpi	14 dpi	Body weight difference at 5 dpi
Control	79 ± 0.58	83 ± 0.58	89 ± 0.58	+4.82
APMV-1	75.8 ± 0.17	71 ± 0.58	74.3 ± 1.20	-6.33
APMV-2	77 ± 1.15	75.3 ± 0.3	79.3 ± 0.67	-2.22
APMV-3	78.3 ± 1.2	77.3 ± 2.69	80 ± 2.89	-1.33
APMV-4	77.6 ± 0.88	80.6 ± 0.67	84.67 ± 0.33	+3.72
APMV-5	82.3 ± 1.45	83.33 ± 1.67	90 ± 1.15	+1.2
APMV-6	77 ± 0.56	72.33 ± 1.45	80.33 ± 0.33	-6.45
APMV-7	77 ± 0.58	72.67 ± 1.78	74 ± 1	-5.96
APMV-8	78.3 ± 1.20	72.33 ± 1.45	75.3 ± 1.45	-8.26
APMV-9	78 ± 1.15	66.33 ± 0.67	80 ± 2.89	-17.59

Mean values with standard error are shown. The values of the difference in body weight at 5 dpi are calculated relative to day 0 for the same group and are expressed in percentage gain (+) or loss (-).

**Figure 1:**
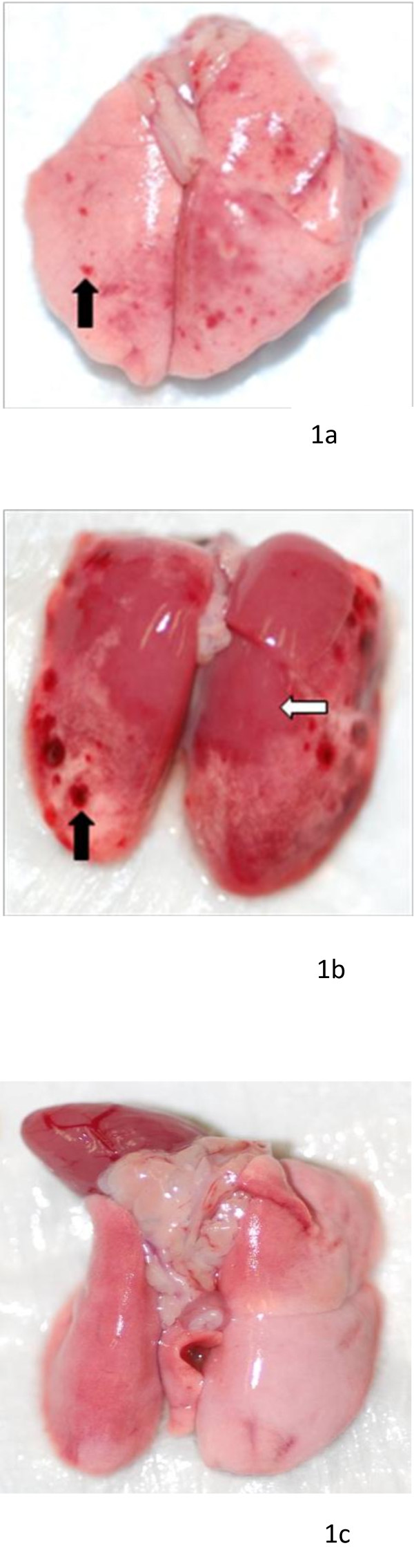
**Gross pathology in lungs from hamsters 3 dpi with APMV-2 (a) and -3 (b)**. (a) Lungs from a hamster infected with APMV-2, showing multifocal mottling with petechial hemorrhages (black arrow). (b) Lungs from a hamster infected with APMV-3, showing multifocal areas of consolidation (white arrow) and ecchymotic hemorrhage (black arrow). (c) Lungs from a mock infected hamster (control).

Following sacrifice on days 3 and 14, the followings organs were removed and examined for gross pathology, histopathology, immunohistochemical analysis, and quantitative virology: brain, lungs, nasal turbinates, small intestine, kidney and spleen. Gross pathologic findings were limited to the lungs of hamsters inoculated with APMV-2 (Figure 1a) and APMV-3 (Figure 1b), and were observed only at 3 dpi. In APMV-2-infected hamsters, the entire pulmonary parenchyma was wet, glistening and edematous, with some areas of reddening due to congestion and hemorrhages. There were several diffuse small round shaped red foci on the lungs. Similar gross pathology was also seen in the lungs of APMV-3 infected hamsters, but with foci that were five times larger than those seen in APMV-2 infected hamsters. No such gross lesions were observed in the hamsters of the other infected groups at 3 dpi. There were no gross visceral pathologic lesions in any of the infected hamsters at 14 dpi. No lesions were detected in the uninfected control hamsters.

### Histopathology

**Figure 2:**
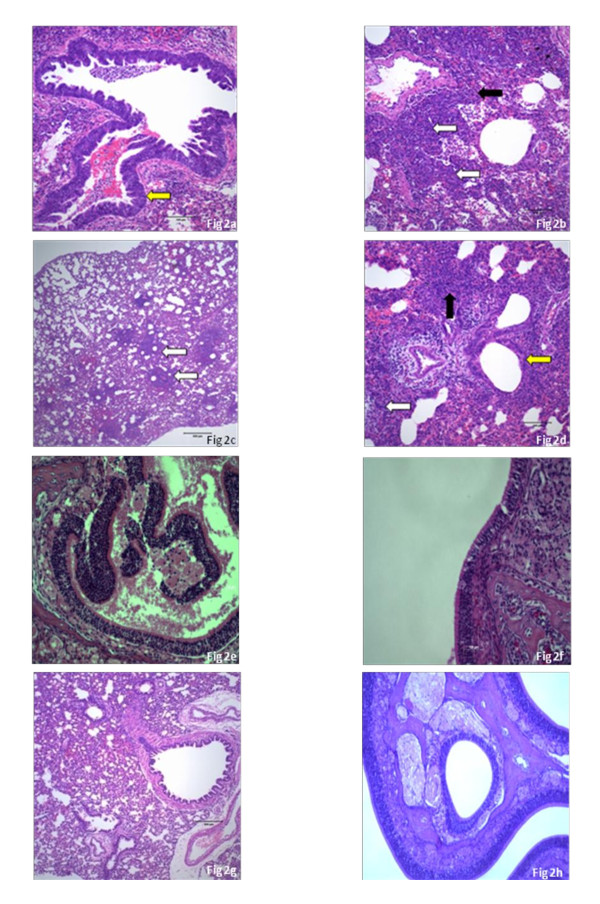
**Representative histopathologic lesions in sections of lung (a-d) and nasal turbinates (e, f) from hamsters 3 dpi with APMV-1 (a, b), APMV-2 (c), APMV-7 (d) and APMV-9 (e, f)**. Lungs (g) and nasal turbinates (h) from uninfected hamster. (hematoxylin and eosin staining, magnification, ×400). (a) Section of lung infected with APMV-1. The bronchiolar and bronchial epithelia are multifocally hyperplastic (yellow arrow) and contain sloughed cellular debris and inflammatory cells (black arrow). There are moderate numbers of necrotic cells in the bronchiolar epithelium and in areas of inflammation in alveoli. (b) Section of lung infected with APMV-1. There is multifocal infiltration and consolidation of the pulmonary interstitium by moderate numbers of lymphocytes, monocytes, and neutrophils (black arrow), with an accumulation of inflammatory cells in alveoli (yellow arrow). Multifocally, alveoli are lined by rows of hyperplastic epithelial cells (white arrows) that sometimes exhibit cellular atypia, including anisocytosis, anisokaryosis, and cytomegaly (atypical type II pneumocyte hyperplasia). (c) Section of lung infected with APMV-2. The pulmonary interstitium is multifocally infiltrated by moderate numbers of lymphocytes, monocytes, and neutrophils (white arrows). (d) Section of lung infected with APMV-7. The pulmonary interstitium is multifocally infiltrated and consolidated by moderate numbers of lymphocytes, monocytes, and neutrophils (black arrow), with accumulations of inflammatory cells in alveoli. Multifocally, alveoli are lined by rows of hyperplastic epithelial cells (white arrow), with some areas exhibiting cellular atypia, including anisocytosis, anisokaryosis, and cytomegaly (atypical type II pneumocyte hyperplasia). The bronchiolar and bronchial epithelia are multifocally hyperplastic (yellow arrow). There are moderate numbers of necrotic cells in bronchiolar epithelium and in areas of inflammation in alveoli. (e) Section of nasal turbinate infected with APMV-9. There is massive cell death and necrosis of the epithelial tissue lining the turbinate bone (white arrow). There is no appreciable level of inflammatory cells in the surrounding tissues. (f) Section of nasal turbinate infected with APMV-3, showing necrotic tissue, loss of ciliary tissue, and blunting of cilia (white arrow). (g) Section of lung from a mock infected hamster (control). (h) Section of nasal turbinate from a mock infected hamster (control).

Histopathological lesions at 3 dpi were observed in the nasal turbinates and lungs in all of the infected groups (Figure 2, and data not shown). Figure 2e and 2f show representative examples of the nasal turbinates for APMV-9 and -3, respectively. The most predominant histopathological lesions observed in the nasal turbinates were multifocal necrotic/apoptotic epithelial cells and nuclear pyknosis. Along the nasal septum were areas of epithelial necrosis, accumulation of nuclear debris in the mucosal epithelium, vacuolation of epithelial cells, and blunting or loss of cilia. Small numbers of mononuclear cells and neutrophils multifocally infiltrated the nasal mucosa, with transcytosis and exocytosis into the nasal cavity. There were small accumulations of inflammatory cells and necrotic cellular debris in the nasal cavity.

Examples of lung tissue are shown for APMV-1 (Figure 2a and 2b), -2 (Figure 2c), and -7 (Figure 2d). All the lung samples from the infected groups exhibited interstitial bronchopneumonia of varying severity at 3 dpi. The inflammatory cell infiltrates were mixed populations of lymphocytes, macrophages, and neutrophils. Additionally, bronchiolar and type II pneumocyte hyperplasia in areas of inflammation with variable degrees of cellular atypia were noticed and the degree of cellular atypia was quite prominent. There were no histopathologic findings for any other organs (brain, small intestine, kidney, and spleen) from any infected hamsters on day 3, or for any of the hamsters at 14 dpi.

### Immunohistochemistry

**Figure 3:**
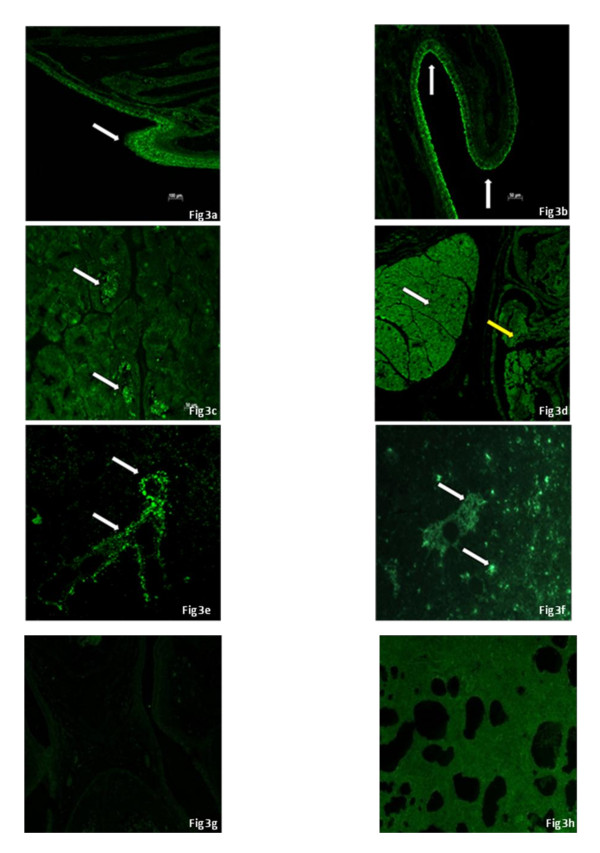
**Immunofluorescence localization of viral N antigen in sections of nasal turbinates (a, b), harderian glands (c, d) and lungs (e, f) from hamsters 3 dpi with APMV-9 (a), APMV-1 (b), APMV-9 (c), APMV-8 (d), APMV-3 (e), and APMV-2 (f)**. Nasal turbinates (g) and lung (h) from a representative uninfected control hamster (magnification, ×400). (a) Section of nasal turbinate infected with APMV-9. Immunofluorescence was evident in the ciliated epithelium lining the turbinate bone (white arrows). (b) Section of nasal turbinate infected with APMV-1. Immunofluorescence was evident primarily at the apical surface of the ciliated epithelial cells and in the cytoplasm (white arrows). (c) Section of harderian gland infected with APMV-9. Immunofluorescence was evident primarily in the collecting ducts of the gland and also in the cytoplasm of the infected cells (white arrows). (d) Section of harderian gland and nasal turbinate infected with APMV-8. Immunofluorescence was evident primarily the cytoplasm of the infected cells in the harderian gland (white arrows) and in the epithelial cells lining the turbinates (yellow arrows). (e) Section of the lung infected with APMV-3. Immunofluorescence was evident around the bronchial epithelium (white arrows). (f) Section of lung infected with APMV-2. Immunofluorescence was mainly evident around the bronchiolar epithelium (white arrows). (g) Section of nasal turbinate from a mock infected hamster (control). (h) Section of lung from a mock infected hamster (control).

Deparaffinized sections of the virus-infected and uninfected control tissue (brain, lungs, nasal turbinates, small intestine, kidney, and spleen) were immunostained using polyclonal antisera against the N protein of the homologous APMV serotypes. Remarkably, animals infected with APMV-5 did not show any positive immunofluorescence in any of the tissues examined at 3 or 14 dpi. In animals infected with any of the other APMV serotypes, virus-specific antigens were detected on 3 dpi in the lungs and nasal turbinates (Figure 3). In the nasal turbinates, virus-positive immunofluorescence was noticed throughout the nasal epithelium lining the turbinate bone, and the harderian gland located near the eye showed complete diffuse fluorescence throughout the organ. In the lungs, the viral antigens were mostly localized in the epithelium surrounding the medium and small bronchi. Viral N antigens were not detected in any additional organs of infected hamsters at 3 dpi, and were not detected in any of the organs of infected hamsters at 14 dpi. The organs of uninfected control hamsters were also negative by immunohistochemistry assay.

### Virus isolation and titration in tissue samples

To analyze sites of virus replication, several organs (brain, lungs, nasal turbinate, small intestine, kidney and spleen) were collected on 3 and 14 dpi, for virus isolation and titration. For all of the APMV serotypes except APMV-5, titration was performed in DF-1 cells and the titers were expressed in TCID_50_/g tissue; for APMV-5, titration was by plaque assay in Vero cells (Table 2). Virus was detected for a number of APMV serotypes on 3 dpi; no virus was detected in any sample for any serotype on 14 dpi. APMV-1 was isolated from lungs and nasal turbinates of all three animals, with mean titers of 1.7 × 10^3 ^and 1 × 10^3^, respectively. APMV-2 was isolated from lungs (3/3 animals) and nasal turbinates (2/3 animals), with mean titers of 4.1 × 10^3 ^and 5.3 × 10^3^, respectively. APMV-3 was isolated from lungs and nasal turbinates of all three animals, with mean titers of 5.3 × 10^2 ^and 3.6 × 10^4^, respectively. APMV-4 was isolated from lungs and nasal turbinates of all three animals, with mean titers of 1.7 × 10^2 ^and 4.2 × 10^3^, respectively, and from the kidneys of one animal (1 × 10^1^). APMV-6 was isolated from the lungs and nasal turbinates of all three animals, with mean titers of 2.6 × 10^3 ^and 7 × 10^1^, respectively, and from the spleen (1 animal, 3 × 10^1^) and small intestine (1 animal, 3 × 10^1^). APMV-9 was isolated from the nasal turbinates only from all three animals, with a mean titer of 2.5 × 10^5^. No virus was isolated from any tissues of the hamsters infected with APMV-5, -7 and -8. Also, no virus was isolated from the brain of any hamsters infected with APMV-1 to -9.

**Table 2:** Virus titers in the indicated tissues from hamsters 3 dpi with APMV serotypes 1 to 9.

Virus	Hamster **No**.	Nasal Turbinates	Lung	Brain	Spleen	Kidney	Small Intestine
APMV-1	1	3 × 10^3^	2 × 10^3^	--	--	--	--
	2	2.4 × 10^3^	3 × 10^3^	--	--	--	--
	3	4 × 10^3^	1 × 10^2^	--	--	--	--
APMV-2	1	9.29 × 10^3^	1.7 × 10^3^	--	--	--	--
	2	3.4 × 10^3^	6.6 × 10^2^	--	--	--	--
	3	3.5 × 10^3^	^-^	--	--	--	--
APMV-3	1	3 × 10^4^	4 × 10^2^	--	--	--	--
	2	4.06 × 10^4^	5.5 × 10^2^	--	--	--	--
	3	3.75 × 10^4^	6.6 × 10^2^	--	--	--	--
APMV-4	1	2 × 10^3^	3 × 10^2^	--	--	--	--
	2	2 × 10^3^	1 × 10^1^	--	--	1 × 10^1^	--
	3	2 × 10^2^	2 × 10^2^	--	--	--	--
APMV-5	1	--	--	--	--	--	--
	2	--	--	--	--	--	--
	3	--	--	--	--	--	--
APMV-6	1	1 × 10^2^	3 × 10^3^	--	--	--	3 × 10^1^
	2	1 × 10^1^	4 × 10^3^	--	--	--	--
	3	1 × 10^2^	1 × 10^3^	--	3 × 10^1^	--	--
APMV-7	1	--	--	--	--	--	--
	2	--	--	--	--	--	--
	3	--	--	--	--	--	--
APMV-8	1	--	--	--	--	--	--
	2	--	--	--	--	--	--
	3	--	--	--	--	--	--
APMV-9	1	3 × 10^5^	--	--	--	--	--
	2	1.5 × 10^5^	--	--	--	--	--
	3	3.8 × 10^5^	--	--	--	--	--

The virus titer values are expressed in TCID_50 _per gram of the indicated tissue sample

### Serology

The virus replication in the hamsters infected with APMVs was further investigated by measuring seroconversion 14 dpi. Sera were analyzed by HI assay using chicken erythrocytes (Table 3) in the case of all of the serotypes except APMV-5, which was analyzed by plaque reduction assay. The HI titers of the pre-infection hamsters were 2 or less. All HI titers greater than 8 were considered positive. Our results showed that all of the infected hamsters seroconverted at 14 dpi, indicating replication by all APMV serotypes. The mean HI titers in hamsters for APMV-1, -2, -3, -4, -6, -7, -8, -9 were 1:256, 1:512, 1:512, 1:32, 1:64, 1:64, 1:1024, 1:256, respectively. The mean serum antibody titer of APMV-5 as determined by virus plaque neutralization test was found to be 1:256. The antigenic relationship among APMV serotypes was evaluated by reciprocal HI tests using these sera, which represent convalescent sera obtained by a single infection of hamsters via the intranasal route. Each of the antisera exhibited very high HI titer against the homologous serotype and either no or very low HI titer against heterologous serotypes, with the exception of APMV -1 and APMV-9 (Table 3). The antiserum specific for APMV-1 and APMV-9 exhibited substantial cross-reactivity, although the APMV-1-specific antiserum had a four-fold higher titer against APMV-1 than against APMV-9, and the APMV-9 specific antiserum had a two-fold higher titer against APMV-9 than against APMV-1. These reactions indicated the existence of antigenic relatedness between APMV-1 and APMV-9.

**Table 3:** Cross-reactivity of sera from hamsters infected with the indicated APMV serotype (top) against the indicated APMV serotype (left column)*.

		**SERUM**								
		**APMV-1**	**APMV-2**	**APMV-3**	**APMV-4**	**APMV-5**	**APMV-6**	**APMV-7**	**APMV-8**	**APMV-9**
	
**VIRUS**	APMV-1	1:256	0	0	0	0	0	0	0	1:128
	APMV-2	0	1:512	0	0	0	0	0	0	0
	APMV-3	0	0	1:512	0	0	0	0	1:8	1:4
	APMV-4	1:8	0	0	1:32	0	0	0	0	1:4
	APMV-5	0	0	0	0	1:256	0	0	0	0
	APMV-6	0	0	0	0	0	1:64	0	0	0
	APMV-7	0	0	0	0	0	0	1:64	0	0
	APMV-8	0	0	0	0	0	0	0	1:1024	0
	APMV-9	1:64	0	0	0	0	0	0	0	1:256

* Sera were harvested 14 dpi. Cross-reactivity was measured by HI assay except when tested against APMV-5, in which case a plaque neutralization assay was used. HI titers represent the highest dilution that displayed HI activity, and are expressed in mean HI units. For APMV-5, the 70% plaque reduction titer is shown as the mean.

## Discussion

APMVs are frequently isolated from a wide variety of avian species and are grouped into nine serotypes based on antigenic reactions [48]. APMV-1 (NDV) is the most extensively characterized member of the APMV serotypes. APMV-2 to -9 have been isolated from both wild and domestic birds, but their disease potential in wild or domestic birds is largely unknown. APMV-1 has also been shown to infect a number of non avian species [41] and presently is being evaluated as a potential human vaccine vector for human pathogens [45]. Therefore, there is a possibility that APMV -2 to -9 could also be used as human vaccine vectors for human pathogens. However, the ability of APMV-2 to -9 to replicate in mammalian hosts was unknown.

Hence, we have investigated the replication and pathogenicity of APMV-1 to -9 in hamsters inoculated intranasally. The intranasal route was intended to resemble the natural route of APMV infection as well as a likely route for vaccine vector administration. In this study, hamsters were chosen as the mammalian animal model because they are widely used to study replication and pathogenesis of a variety of viruses such as adenovirus [49], herpes virus [50], Nipah virus [51], human RSV [52], human parainfluenza viruses, SARS virus [53], and Eastern equine encephalitis [54].

Clinical disease was not evident with most of the APMV serotypes, and was observed only in the case of APMV-9. All of the animals infected with APMV-9 exhibited clinical disease including weakness and substantial weight loss. Only APMV-2 and -3 produced gross pathological lesions in the lungs, whereas in hamsters infected with the other APMV serotypes the appearance of the lungs was unremarkable. No gross pathology was evident for any other organs with any of the serotypes.

Virus was recovered from animals infected with APMV-1, -2, -3, -4, -6, and -9. In all cases, virus was isolated 3 dpi: no virus was detected in any samples for any serotype 14 dpi. The viral titers were moderate and were mostly restricted to the lungs and nasal turbinates; there were isolated incidences of isolation from the spleen (APMV-6), small intestine (APMV-6), or kidneys (APMV-4). No virus was recovered from any tissue from animals infected with APMV-5, -7 and -8. However, the lungs and nasal turbinate tissues from animals infected with APMV-7 and -8 were positive by IHC, indicating that these viruses infected and replicated at a level that was below detection by our assays for quantitative virology. Although APMV-5 was not detected by virus isolation or by IHC in any of the tissues, all of the animals that were inoculated with APMV-5 developed titers of APMV-5 specific antibodies detected by virus plaque reduction neutralization assay, indicating a low level of replication. By 14 dpi, no virus could be detected in any of the tissues in any the infected hamsters for any serotype, whether by histopathology, histochemistry, or virus isolation. This suggests that the virus was cleared from all tissues and disease was resolved, indicating the self-limited nature of the infections. Similar results have been obtained from chickens infected with APMV-2, -3 and -4, where no virus could be isolated at 14 dpi [30].

Using IHC, viral N antigen was detected in the same tissues that were positive by virus isolation. An interesting finding was the presence of large amounts of viral antigens at the epithelial cell linings, suggesting that these cells are highly permissive to APMV replication. In addition, the detection of viral antigens, and in most cases infectious virus, in nasal turbinates and lungs of the hamsters indicate that APMV replication is mostly restricted to the respiratory tract. These results show that all APMV serotypes are capable of infecting hamsters using a nasal route of infection and the extensive amount of virus replication in the respiratory tract was not accompanied by severe disease in hamsters.

Serologic assays demonstrated a humoral response in all the hamsters inoculated with APMV serotypes -1 to -9, a further indication of successful virus replication. Our results show that all APMVs (except APMV-5, which does not hemagglutinate) produced HI antibody titers that varied between 1:32 to 1:1024. Based on the HI titers, APMV-8 (1:1024) produced maximum antibody titer and APMV-4 (1:32) produced the least. Paradoxically, while APMV-8 induced the highest titer of HI antibodies, the virus could not be isolated from any of the infected hamsters. Conversely, APMV-4 replicated efficiently in hamsters as observed by virus isolation in nasal turbinates, lungs and kidneys, but produced low levels of virus-specific HI antibodies. Whether these examples of incongruity between levels of replication and serum antibody responses are indicative of differences in the antigenicity of the respective HN proteins or to some other factor remains to be determined.

Warke et al. [30] have also provided evidence of low HI titers in the case of APMV-4, in chickens, which was taken as evidence of a low level of replication of this virus in chickens. The HI test is the most commonly used method to diagnose APMV infections and also is used to measure the antibody response. But chicken antiserum against NDV cross-reacts in HI tests with several of the other APMV serotypes, thus questioning the specificity of HI test in field cases [55]. Warke et al. [38] indicated that the HI test lacks sensitivity for detecting infection with these APMV serotypes. They also observed that even in the case of a high infectious dose and observed microscopic changes in the infected organs, high HI titers were not observed in the infected birds. On the contrary in our study, most of the APMV serotypes replicated well in hamsters, producing mild respiratory pathology and high neutralizing antibody titers. Our results indicated that the antibody developed in hamsters against these serotypes were very specific and no cross reaction was observed between serotypes except between APMV-1 and -9. Cross-reactivity between these two serotypes is not completely unanticipated, since they share the highest level of genome nucleotide sequence identity (58%) among the APMV serotypes [12]. Hence, we conclude that the HI test with hamster serum is highly specific and can be used to diagnose different APMV serotypes in field cases.

The replication of APMVs in hamsters produced mild rhinitis and mild pathology that was mainly restricted to the respiratory tract. There was a concern that APMVs may also replicate in the intestine and shed in feces, which might act as a source of infection for the other animals. But our results indicated that none of the APMVs replicate in the intestinal epithelial cells of hamsters. Importantly, our results also suggest that these viruses do not cross the blood-brain barrier and do not induce neurological symptoms. Also, our previous experimental studies of all the APMVs in chickens showed that they were avirulent. Taken together, these results show that the APMVs replicate moderately in hamsters and produce either mild or no clinical signs, and elicit substantial antibody responses. Therefore, it is possible that APMVs might replicate in other mammalian species including humans. In conclusion, this study is the first comparative report on the replication and pathogenicity of prototype strains of all 9 APMV serotypes in hamsters. Our results lay the foundation for a good laboratory animal model for testing the replication and pathogenicity of APMV strains.

## Competing interests

The authors declare that they have no competing interests.

## Authors' contributions

AS carried out the pathogenesis studies, carried out the immunoassays, performed the analysis, and drafted the manuscript. MS carried out the pathogenesis studies. HS participated in the analysis, interpretation of histopathological slides. PC and SKS, conceived the study, participated in its design and coordination. All authors read and approved the final manuscript.
